# How (Eco)immunology can augment global EcoHealth programmes: opportunities, needs, and challenges

**DOI:** 10.1093/discim/kyae015

**Published:** 2024-10-15

**Authors:** Sheena M Cruickshank, Kathryn J Else, Iris Mair, Holly Shiels, Susanne Shultz

**Affiliations:** School of Biological Sciences, Faculty of Biology, Medicine and Health, Lydia Becker Institute of Immunology and Inflammation, University of Manchester, Manchester, UK; School of Biological Sciences, Faculty of Biology, Medicine and Health, Lydia Becker Institute of Immunology and Inflammation, University of Manchester, Manchester, UK; School of Biological Sciences, College of Science and Engineering, Institute of Ecology and Evolution, Institute of Immunology and Infection Research, University of Edinburgh, Edinburgh, UK; School of Earth and Environmental Sciences, Faculty of Science and Engineering, Manchester Environmental Research Institute, University of Manchester, Manchester, UK; School of Earth and Environmental Sciences, Faculty of Science and Engineering, Manchester Environmental Research Institute, University of Manchester, Manchester, UK

**Keywords:** EcoHealth, interdisciplinary research, immunology, ecology, ecoimmunology

## Abstract

**Graphical Abstract:**
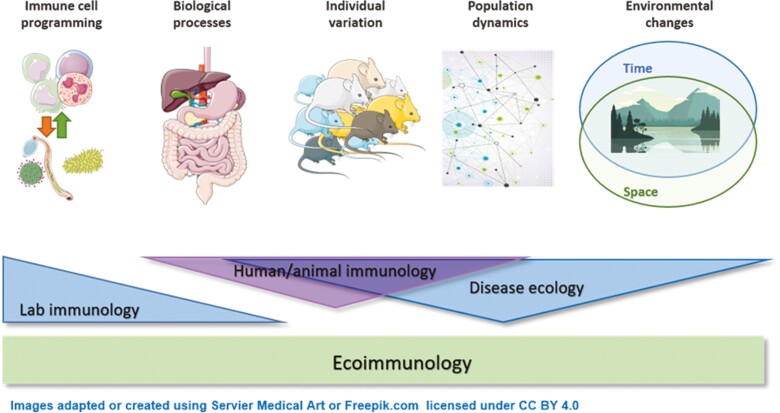

Accelerating human footprints on the planet, including climate change [1], pollution [2], and changes in land use [3], impact immune function, drive non-communicable, and communicable diseases, create exposure to zoonotic conditions, and introduce novel pathogens. We urgently need to understand the differential effects of a changing environment on health and how this contributes to health inequalities. An EcoHealth approach captures the link between the environment and health and enables a holistic debate around communicable and non-communicable diseases. The interdisciplinary field of ecoimmunology can accelerate EcoHealth programmes. Our recommendations, arising from an ecoimmunology workshop held at the University of Manchester, transcend ecoimmunology and are relevant to all areas of interdisciplinary research.

A competent immune system is critical for health as it protects against a range of infectious agents whilst discriminating against beneficial things such as food and the microbiome. Consequently, our immune system is malleable. The cumulative environmental conditions, which we face throughout our lives and our exposome, shape our immune state. From birth, extrinsic factors, including lifestyle factors such as diet [4, 5], pollutants [6, 7], and infections [8, 9], will influence our immune system’s balance towards, for example, a pro-inflammatory or anti-inflammatory state, tipping the balance towards allergy, autoimmunity, or susceptibility to infection.

At the forefront of immunological research for decades, the laboratory mouse has played and will continue to play, a major role in immunological research. Indeed, laboratory mouse models of infectious and non-communicable diseases have contributed significantly to understanding the immune mechanisms underlying host responses to disease, facilitated by the high level of control that can be applied to variables of interest. However, laboratory animals are kept in highly sanitized, controlled conditions and consequently, have profoundly different immune responses to their wild counterparts. Indeed, the immune system of a naive laboratory mouse has some features more akin to the immune system of a newborn baby than that of an adult human, with, for example, differences in the abundance and phenotype of CD8+ T cells [10]. The Hygiene Hypothesis [11] and Old Friend’s Hypothesis [12] also recognize the importance of environment and infection in immune-mediated disease. Paradoxically, the strengths of lab-based and ecoimmunological studies are also their limitations. Ecoimmunology tries to ascribe causal roles from studies where many key variables cannot be constrained, or where they may even be unknown. Lab-based studies are limited by an inability to embrace multiple variables. Collectively, ecoimmunology studies can inform lab-based studies and vice versa. There is thus an exciting space for combining the strengths of both ‘traditional’ and ecoimmunology approaches to better model different environments capturing the complexities of context-dependent exposure. Disease outcomes are undoubtedly dictated by the quality of the immune response; understanding how the immune system operates in a real-world context is crucial for determining the drivers of susceptibility to infection, inflammation, lifespan, and quality of life. The exposome contributions to immune variation likely result from a complex network of influencing factors that are challenging to model in the lab and which can only be explored in natural ecologically relevant systems [13, 14].

Ecoimmunology brings together two long-established and seemingly opposing disciplines, ecology with immunology. Ecologists explore uncontrolled variation in a population using epidemiological, ecological, and mathematical tools. Immunologists tend to minimize variation to understand cellular mechanisms at play in individuals. The intersection of the two disciplines is ecoimmunology (Figure 1). For example, some immunologists have been ‘dirtying’ up their model species, for example, by co-housing laboratory mice with pet shop mice [15], transferring microbiomes from wild mice into germ-free laboratory mice [16], and transplanting laboratory mouse embryos into wild mouse recipients [17]. Furthermore, laboratory mice exposed to wild mouse microbiomes have been shown to respond more akin to humans when challenged with drugs that failed in clinical trials than their clean laboratory counterparts [17], highlighting the potential translational power of ‘dirtier’ model systems. Equally, ecologists studying wild animal species have started incorporating immunological questions. Ecoimmunology offers a conceptual approach for studying immunology in our multivariate real world, providing much-needed context-dependent model systems. Any model of ecoimmunology must account for how environmental and social challenges of our interconnected world impact health and immune function. Such interdisciplinary approaches will facilitate new discoveries allowing us to better understand how extrinsic variables shape our immune system.

**Figure 1.**
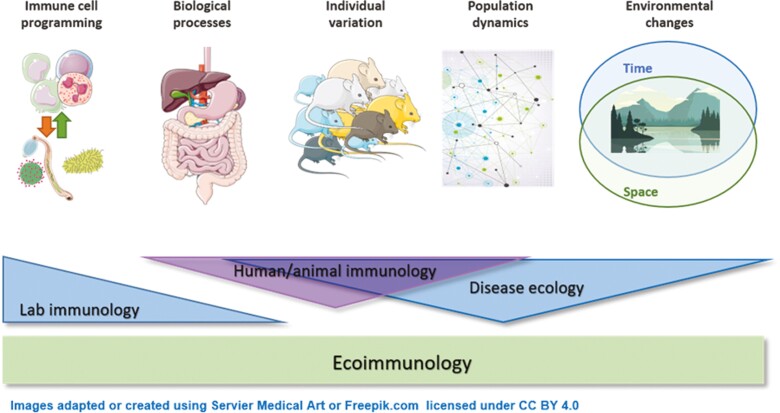
Ecoimmunology. Fundamental discovery lab-based immunology typically focuses on defined model systems—(*in vitro* or *in vivo* models such as mice or *ex vivo* tissues) to understand immune programming in the context of biological processes, health, and disease. In contrast, disease ecology focuses on the role of the environment and considers population dynamics and individual variation. Whilst there is some limited overlap in disciplines, the area of ecoimmunology aims to bridge these gaps and takes a more holistic approach to understanding what shapes the immune system and thereby health.

## The challenges that can be explored through an ecoimmunology lens

### Climate change

Climate change presents direct and indirect challenges to the immune system. Intense heat is linked to heat stroke, cardiovascular and respiratory distress, and preterm births. Prolonged heat can lead to drought affecting crops and animal die-offs. Temperature also modulates immune function with even mild hyperthermia (e.g. 38.5°C) significantly reducing pro-inflammatory cytokine secretion, which then impacts immune efficacy [18]. Increased humidity drives an increased risk of floods, which increases exposure to contaminants and infectious agents threatening health and crop biosecurity. Food insecurity adds to the risks of malnutrition, which causes secondary immunodeficiency [19]. Altered climates particularly enhance the potential for breeding of vectors resulting in enhanced transmission of vector-borne diseases such as dengue [20]. The distribution of diseases, such as leishmaniasis and schistosomiasis, is changing, with the incidence predicted to increase [21]. Climate change-related illnesses disproportionately impact disadvantaged communities and more vulnerable groups such as the elderly. Moreover, prior inequalities such as malnutrition and/or environmental contaminant exposure are compounded by climate change pressures driving greater division in health and immune function across all life stages emphasizing the cyclic and often amplifying effects of our interconnected world.

### Changing ecosystems

In addition to the direct consequences of climate change, other human activities affecting land use drive drastic changes in global ecosystems. Deforestation [22] due to intensification of agriculture can enhance human–livestock interactions facilitating zoonotic infection transmission. Equally, as land use changes due to pressures of reduced grazing land, extensive mixing of wildlife and livestock occurs bringing the risk of emerging infectious diseases such as SARS and Ebola. Notably, most of the recent epidemics and pandemics are due to zoonotic infections via direct transmission or indirectly via vectors [23]. Urbanization as well as greater international travel and trade enhance the spread of infectious diseases and invasive species. Invasive species can threaten biodiversity and food supply, further risking food security and infection transmission. Intensive farming practices also contribute to antimicrobial resistance [24] and can drive pollution of water sources [25]. In addition to the threats of novel infections, altered habitats and habitat loss impose stress—an important modulator of the immune system linked with immune dysfunction [19, 26].

### Pollution

Pollution has multiple impacts on plant, animal, and human health [2]. Pollution causes morbidity directly and by worsening pre-existing conditions such as heart disease, stroke, chronic obstructive pulmonary disease, asthma, and acute respiratory infections such as coronavirus disease 2019 (COVID-19) and influenza [27, 28]. Forever chemicals such as per- and poly-fluoroalkyl substances used in making non-stick materials can build up in an organism over time (bioaccumulate) and become more concentrated as they move up through the food chain (biomagnify) thereby impacting health, disease resistance, and fecundity. Increased and intensified agriculture and enhanced use of pesticides is a profound threat to pollinators which, in turn, impacts our food biosecurity [29]. Akin to climate change, the impact of pollution on health is unequal, disproportionately impacting vulnerable groups. Indoor pollution also has a huge impact on health and poor stock housing and damp conditions can favour mould production which, in turn, drives fungal infection and asthma [30]. Mechanistically, pollution damages protective cellular barriers enhancing susceptibility to infectious agents and altering immune cell function, for example, phagocytic capacity. Pollution can also have direct impacts on infection transmission with, for example, changes in cell receptor expression enabling enhanced colonization by viruses.

To contextualize immunological research and tackle all these multifactorial challenges requires a holistic approach crossing disciplines, but many challenges remain in place that prevent us from successfully bridging [31]. We held a multidisciplinary workshop on ecoimmunology, including ecologists, immunologists, social scientists, economists, mathematical modellers, physicists, and public health scientists. We asked if we, as individual scientists and research communities, were equipped to meet the growing threats to our health and made recommendations for how we can move forward. The outcomes of this workshop are summarized in Box 1.

## Recognizing and capitalizing on the opportunities of multidisciplinary research in immunology

There is an increasing need for interdisciplinary research to tackle some of the biggest issues our society is facing. Building critical mass in interdisciplinary research in immunology requires the facilitated engagement of people from diverse disciplines and backgrounds including community groups, patients, businesses, and policymakers. The sheer global relevance of all the themes discussed in this paper heightens the urgency for research and the need for low-middle-income-country (LMIC) involvement in these areas.

The COVID-19 pandemic illustrated the need for interdisciplinarity. The academic immunology research community [32, 33] was a vital part of the effort in the development of vaccines and therapies, but this required input from patient groups as well as diverse researchers. In the pandemic, we also saw how we could adapt our systems to enhance trials for vaccines and drugs. However, the pandemic also showed us the threat of miscommunication, misunderstanding, and the fragility of trust.

To conclude, we highlight the growing risk of communicable and non-communicable diseases in a world of our creating on immune function. We make several recommendations to overcome the barriers that are slowing progress in our understanding of the differential effects of a changing environment on health and well-being within an EcoHealth and ecoimmunology context. The need is clear, the will is there, and we must act to address these global challenges.

BOX 1 **Current Challenges and Recommendations for Solutions**

**Breaking the language barriers at grass roots:**

- Offering multidisciplinary courses, cross-disciplinary units, continuing professional development programmes, and interdisciplinary PhD schemes to breakdown language and methodological barriers.

**Raising awareness of interdisciplinary research and discipline-specific values:**

- Ensure that collectively referees represent each of the scientific disciplines contributing to the interdisciplinary grant application or research manuscript.
- Increase training provision for referees of grants and manuscripts in the formulation of grant reviews.
- Develop more high-impact multidisciplinary journals.
- Increase awareness of the different research disciplines’ view of authorship and the meaning of authorship order: authorship and perceived seniority can influence promotion decisions; however, in some disciplines authors are assigned alphabetically, and senior author position may consequently not be the last author. It is, therefore, important that contributions are credited using, that is, CRediT acknowledgments. Given that interdisciplinary research involves teamwork and thus by definition both multi-author and cross-disciplinary authorships, raising awareness of these authorship differences in values is helpful.

**Consideration of the research infrastructure:**

- Planning to generate the appropriate scale of data, appropriate controls, and conditions.
- Collation and banking of appropriate resources, for example, tissue or soil samples in the correct ways.
- Agreements for data sharing, ethics, and standardization across disciplines and institutions

**Co-creating critical mass at the point of need:**

- Work with LMIC to co-create projects, bring in wider knowledge, and create a more equitable research environment.
- Bring in a diverse public, including policymakers, community groups, patients, and businesses to ensure a variety of expertise and lived experience is considered.
- To establish a UK-wide Ecoimmunology network with membership across fields and spread geographically.

## Data Availability

None declared.
